# Berberine alleviates ox-LDL induced inflammatory factors by up-regulation of autophagy via AMPK/mTOR signaling pathway

**DOI:** 10.1186/s12967-015-0450-z

**Published:** 2015-03-15

**Authors:** Xiaodi Fan, Jun Wang, Jincai Hou, Chengren Lin, Alan Bensoussan, Dennis Chang, Jianxun Liu, Bing Wang

**Affiliations:** Department of Tianjin, University of Traditional Chinese Medicine, Tianjin, 300193 China; Department of Experimental Research Center, Xiyuan Hospital of China Academy of Chinese Medical Sciences, 1 Xiyuan Caochang, Hai Dian District, Beijing, 100091 China; Institute of Basic Theory, China Academy of Chinese Medical Sciences, Beijing, 100700 China; Centre for Complementary Medicine Research, School of Science and Health, University of Western Sydney, Parramatta, NSW 2751 Australia; Department of Anesthesiology and Critical Care Medicine, Johns Hopkins University School of Medicine, Baltimore, MD 21205 USA

**Keywords:** Berberine, Macrophage, Autophagy, Inflammation, AMPK/mTOR

## Abstract

**Background:**

Inflammation induced by oxidized low-density lipoprotein (ox-LDL) plays an important role in the pathogenesis of atherosclerosis. Recently, roles of autophagy against inflammation in the process of atherosclerosis have drawn increasing attention. Here, we tested the possible molecular mechanisms by which berberine confers an anti-inflammatory effect in macrophages by upregulation of autophagy.

**Methods:**

J774A.1 macrophages were incubated with various doses of ox-LDL for various times. We evaluated the inflammatory factors and autophagy proteins (LC3II/LC3I, and SQSTM1/p62) to ascertain the optimal dose and time. Ox-LDL–induced inflammatory factors and autophagy in J774A.1 cells were tested by the AimPlex multiplex assay, Western blotting, confocal microscopy, and transmission electron microscopy in the presence of berberine or chloroquine (CQ). Adenosine 5’-monophosphate-activated protein kinase (AMPK) inhibitor compound C was used to evaluate the AMPK/mTOR signaling pathway.

**Results:**

Berberine dose- and time-dependently reduced ox-LDL–induced inflammation and increased the ratio of LC3II/LC3I, and SQSTM1/p62 in J774A.1 cells. CQ significantly attenuated the berberine-induced autophagy and anti-inflammation. In addition, berberine increased the ratio of p-AMPK/AMPK and decreased the ratio of p-mTOR/mTOR. AMPK inhibitor compound C abolished berberine-induced autophagy and promoted p-mTOR/mTOR expression in J774A.1 cells.

**Conclusion:**

Berberine treatment inhibits inflammation in J774A.1 cells by inducing autophagy, which is mediated through activation of the AMPK/mTOR signaling pathway. Importantly, this study provides new insight into berberine’s molecular mechanism and its therapeutic potential in the treatment of atherosclerosis.

**Electronic supplementary material:**

The online version of this article (doi:10.1186/s12967-015-0450-z) contains supplementary material, which is available to authorized users.

## Background

Atherosclerosis (AS), an imbalance in lipid metabolism and a maladaptive inflammatory response, has been evidenced to be a chronic inflammatory disease of the arterial wall [1]. Oxidized low-density lipoprotein (ox-LDL) is a potential inducer of chemokines, which is recognized as a critical cardiovascular risk [2,3]. Emerging data have demonstrated that modified lipids engulfed by monocyte-derived macrophages resulted in the secretion of pro-inflammatory cytokines and further macrophages recruitment. This contributes to robust increases in atherosclerotic plaque size and complexity [4]. Therefore, understanding the regulatory mechanisms of inflammation in monocytes/macrophages is important for prevention of AS.

Autophagy is the process in which protein aggregates and damaged organelles are removed for the maintenance of intracellular homeostasis during various cell stresses [5]. Recent studies have highlighted the importance of autophagy role in the formation of atherosclerosis. It was demonstrated that autophagy in macrophages plays a protective role in advanced atherosclerosis [6]. Meanwhile, autophagy dysfunction in macrophages leads to the inflammation and thus accelerates atherosclerotic progression [7]. Although how autophagy induction affects pathological processes, such as inflammation, remains to be elucidated, autophagy might be a novel therapeutic strategy for the prevention and treatment of AS [8]. The enhancement of autophagy in macrophages by drugs or other methods potentially inhibits the progression of atherosclerotic plaques and reduces its stability [9]. Some pharmacological agents have been identified for modulation of autophagy in AS. The most common inducer is mTOR inhibitors, such as rapamycin, and its analogs everolimus. Nowadays, some active components from natural medicines have been regarded as the focus in the treatment of AS, such as salvianolic acid B, baicalin, tectoridin, etc.

Berberine (BBR) extracted from *Coptis*, one of the isoquinoline quaternary alkaloid, has a definite potential in clinical because of its diverse pharmacological properties, such as antimicrobial, antidiabetic, antihyperlipidemic, anti-inflammatory, and antioxidant [10,11]. Studies have focused on the therapeutic role of BBR in cardiovascular diseases, especially in coronary heart disease [12]. Additionally, berberine could trigger autophagy in cancer cells, and its beneficial effects were alleviated when the autophagy process was genetically or pharmacologically inactivated, which suggested that autophagy is indispensable for the protective effects of berberine [13,14]. However, the molecular targets by which berberine may exert its beneficial effects have not been fully elucidated. Therefore, we aim to investigate whether berberine could induce autophagy to inhibit secretion of inflammatory factors stimulated by ox-LDL in J774A.1 through AMPK/mTOR signaling pathway.

## Materials and methods

### Cell culture

The murine cell line J774A.1 were obtained from Institute of Basic Medical Sciences of Chinese Academy of Medical Sciences & Cell Resource Center (ATCC Number:CRL-1592). Cells were grown in Dulbecco’s modified Eagle’s medium (DMEM) (Hyclone, SH30022.01B) supplemented with 10% fetal bovine serum (Gibco, 10099-141-FBS), 4 mmol/L glutamine, and 100 U/mL penicillin-100 μg/mL streptomycin. Cultures were maintained at 37°C in a humidified 5% CO_2_ incubator. Cells were identified by their typical cobblestone morphology under microscope.

### Cell treatment

All experiments were performed in complete culture medium to avoid the induction of autophagy via the serum starvation pathway. J774A.1 derived macrophages were exposed to ox-LDL (100 mg/L, Yiyuan biotech, China) for 1 h. Meanwhile, cells were treated with the different concentrations of berberine (110713–201212, National Institutes for Food and Drug Control, Beijing, China) for 24 h or berberine (25 μM) for the different time points without ox-LDL. Cells were also treated with chloroquine (CQ, 30 μM, c6628, Sigma-Aldrich, USA), Compound C (CC, 40 μM, sc200689, Santa Cruz Biotechnology, USA), rapamycin (RAP, 100nM, R0395, Sigma-Aldrich, USA), l-Leucine (LEU, 5 mM, L8000, Sigma-Aldrich, USA) for 1 h following the addition of berberine for 24 h.

### Oil red O staining

After treated by ox-LDL, cells were fixed with 10% formalin, followed by rinse with 60% isopropanol and incubation with fresh-filtered 0.5% oil red O solution for 10 min at 37°C. For analysis, the cells were washed in isopropanol for 10 min, rinsed in distilled water, and hematoxylin was introduced to label the cell nuclei. Images of cells were captured using a fluorescence microscope to evaluate the characteristic lipid accumulation in macrophage-derived foam cells. Foam cell formation was observed under a microscope.

### Immunofluorescence microscopy

To assess the alteration and location of target protein, J774A.1 cells were incubated with anti-LC3 antibody and anti-p62 antibody (both in 1:500), followed by the incubation with corresponding secondary antibodies (1:400), goat anti-rabbit IgG/Alexa Fluor®594 antibody (Invitrogen, USA) conjugated to LC3 and goat anti-rabbit IgG/Alexa Fluor®488 antibody (Invitrogen, USA) conjugated to SQSTM1/P62. Cells were then incubated with 4’-6-diamidino-2-phenylindole (DAPI, Vector Lab) to display nucleus. Finally, cells were analyzed with a confocal microscope (ZEISS, Germany). Images were digitally analyzed using ZEN microsystem software to observe the fluorescence intensity of cells.

### Transmission Electron Microscopy

Cells were fixed with 4% glutaraldehyde and post-fixed in 1% osmium tetroxide at 4°C. The samples were then washed again, dehydrated with graded alcohol, and embedded in Epon-Araldite resin. 50 nm of ultrathin sections were obtained using an ultramicrotome (Leica). Sections were then stained with uranyl acetate and lead citrate. Hitachi H-7500 transmission electron microscope was used to observe autophagosome.

### Bead based multiplex flow cytometry

Aimplex Mouse Inflammation 17-Plex assay kits (Beijing Quantobio, China, C282217) were used according to the manufacturer’s instruction manual. Briefly, the assay procedure consists of a 60 min antigen and capture antibody conjugated bead incubation step, a 30 min biotinylated detection incubation step and a 20 min streptavidin-PE incubation step. Fluorescence signals of the sample beads were acquired by a flow cytometer and the inflammatory factors were analyzed with FCAP Array 3.0.

### Western blotting analysis

Total protein in the supernatant was measured using bicinchoninic acid (BCA) protein assay kit (P0010-1, Beyotime, China). Lysates taken from each sample were separated by 12.5% SDS-PAGE. Blots were probed with 1:1500-diluted primary antibodies against LC3 and SQSTM1/p62 (L7543 and P0067, Sigma-Aldrich, St. Louis., MO, USA), AMPKα, p-AMPKα (Thr172), mTOR and p-mTOR (Ser2448) (5831, 2535, 72949 and 5536, Cell Signaling Technology, USA) over night at 4°C, followed by horseradish peroxidase (HRP)-conjugated secondary antibodies for 90 min at room temperature. Then, the proteins were visualized by enhanced chemiluminescence exposure to Molecular Imager®Gel DocTMXR+ and ChemiDocTMXRS+ Systems. Finally, the blots were scanned, and densitometric analysis was performed on the scanned images using with Image LabTM Software.

### Statistical analysis

Results were expressed as mean ± SEM. Statistical significance between groups was assessed by one-way analysis of variance (ANOVA) followed by LSD (Least Significant Difference) post-hoc test using SPSS Inc. Tamhane post-hoc test was performed if the variances were unequal. P < 0.05 or P < 0.01 was considered statistically significant.

## Results

### The optimal concentration of ox-LDL increased MIP-1α and RANTES, and decreased IL-10 in J774A.1 cells

**Figure 1:**
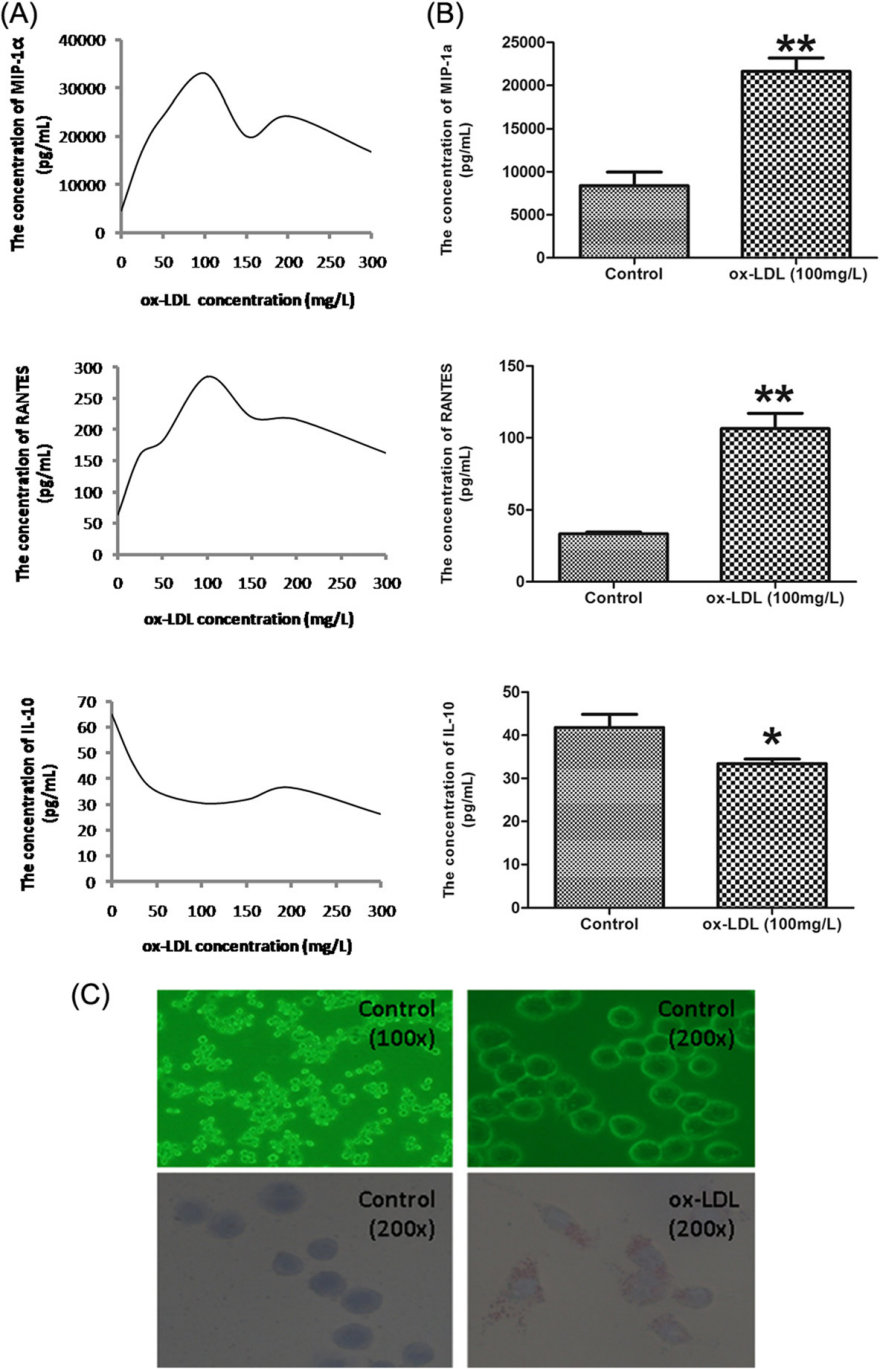
**The changes of inflammatory factors induced by different concentrations of ox-LDL. (A)** Cells were incubated with different concentrations of ox-LDL (0, 25, 50, 100, 150, 200 and 300 mg/L) for 24 h. The levels of MIP-1a, RANTES and IL-10 in the medium were detected via AimPlex. Points and connecting line graphs showed the quantification of the inflammatory factors. From the curvilinear trend, 100 mg/ L ox-LDL was the optimal concentration induced the inflammatory factors. **(B)** Bar graphs showed that 100 mg/L ox-LDL increased the levels of MIP-1a and RANTES, and decreased the level of IL-10. Values are mean ± SEM. (n = 6). **(C)** The morphology of J774A.1 cells under normal culture condition (100×; 200×) or treated with 100 mg/L ox-LDL for 24 h. Oil Red O stainting were used to test the foam cell formation (200×). **P < 0.01 V.S. control group; *P < 0.05 V.S. control group.

To explore the appropriate concentration of ox-LDL at which could induce inflammatory reaction, we detected the level of inflammatory factors induced by a series of concentrations of ox-LDL. Cells were treated with different concentrations (0, 25, 50, 100, 150, 200, 300 mg/L) of ox-LDL for 24 h to induce inflammatory factors. Figure 1A-B showed that 100 mg/L ox-LDL obviously increased MIP-1α and RANTES, and decreased IL-10, compared with other concentrations. Therefore, we chose 100 mg/L ox-LDL as the optimal concentration for the subsequent experiments. The flow cytometry scatter plot of the different concentrations of ox-LDL was shown in Additional file 1: Figure S1. Meanwhile, Oil red O staining indicated that J774A.1 cells were induced to foam cells by 100 mg/L ox-LDL (Figure 1C). Furthermore, we are intended to observe the effect of ox-LDL on autophagy in macrophages.

### Autophagy suppressed inflammatory factors induced by ox-LDL in J774A.1 cells

**Figure 2:**
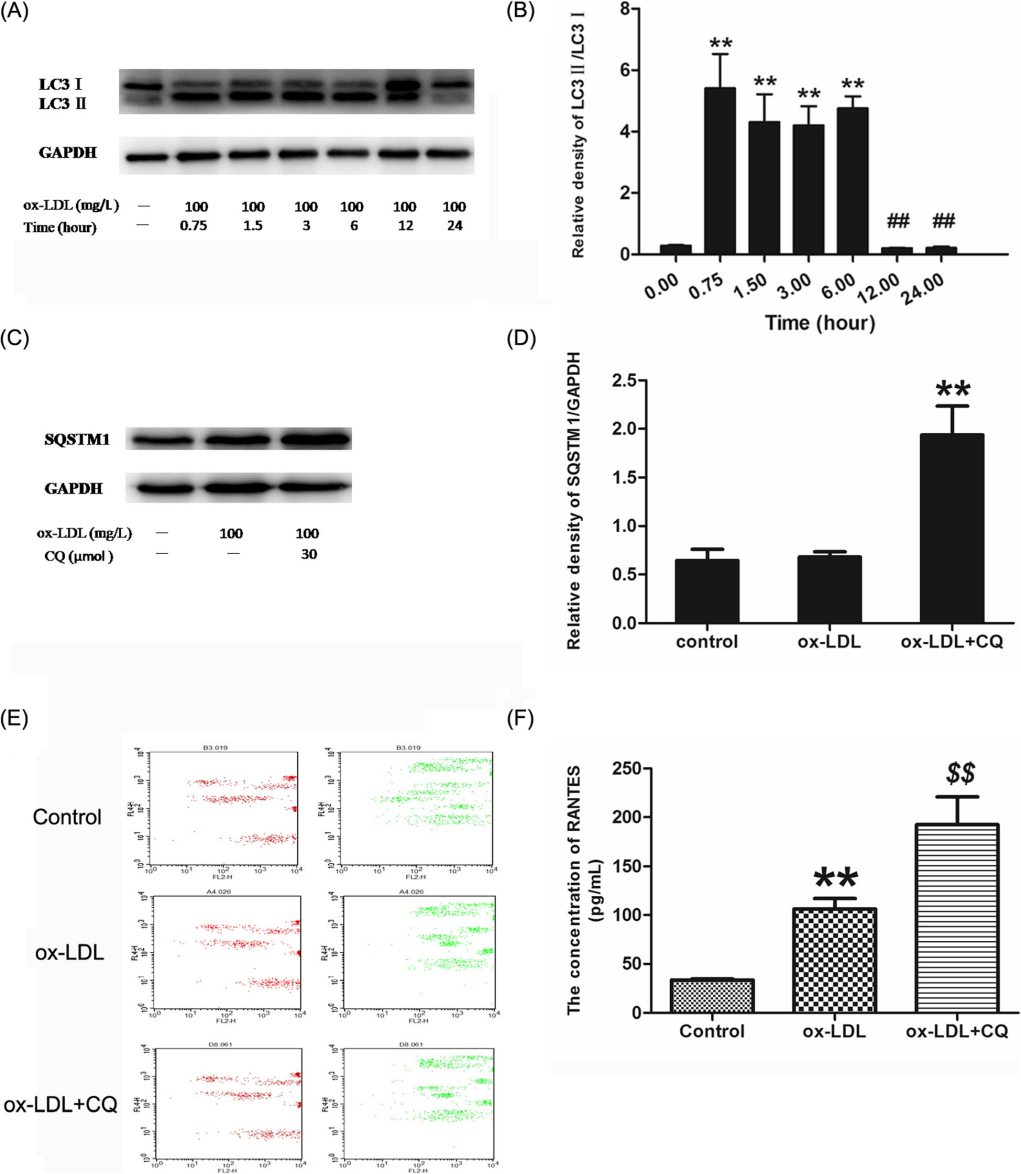
**Endogenous LC3 in the lysates was recognized by immunoblotting with an anti-LC3 antibody. LC3-I, soluble form of LC3; LC3II, membrane-bound form of LC3. (A)** Representative blots of LC3Iand LC3II in J774a.1 cells incubated with 100 mg/L ox-LDL for different time points (0.75, 1.5, 3, 6, 12 and 24 h) using Western blot analysis. **(B)** Bar graphs showed the LC3II/ LC3Iratio quantified by Image Lab 3.0 software. **(C)** Representative blots of SQSTM1 in the cells incubated by ox-LDL in the presence or absence of 30 μM CQ. **(D)** Bar graphs showed the band value of SQSTM1/p62. **(E)** Representative Flow cytometry scatter plot of J774A.1 cells after treatment with ox-LDL and CQ**. (F)** The levels of RANTES in the culture medium were detected via AimPlex. Each experiment was repeated at least three times. **P < 0.01 V.S. control group; ##P < 0.01 V.S. 0.75 h, 1.5 h, 3 h and 6 h groups; $P < 0.05 V.S. ox-LDL group.

To demonstrate whether inflammatory factors could be inhibited by autophagy, we firstly selected different time points (0.75, 1.5, 3, 6, 12, and 24 h) to observe the effect of ox-LDL on autophagy induction in J774A.1 cells. The results showed that ox-LDL increased the expression of LC3, an indicator of autophagy, in J774A.1 cells, at 0.75, 1.5, 3, and 6 h. Per contra incubation with ox-LDL for 12 h or 24 h declined autophagic activity sharply to the basal level (Figure 2A-B). For observation of the effect of berberine on macrophages autophagy, we need to exclude the basal autophagic effect of ox-LDL on macrophages. Considering that 24 h incubation of ox-LDL on macrophages could both increase inflammatory cytokines and reduce the autophagy level to the base level, we used 24 h as ox-LDL incubating time in the following experiments.

Cells were pretreated with CQ (30 μM, a common autophagy inhibitor) for 1 h and then exposed to ox-LDL for 24 h. We found that the expression of LC3 could not be inhibited by 3-MA, an autophagy inhibitor which selectively blocked autophagy during fusion of autophagic vacuoles with lysosomes. However, CQ prevented autophagy by blocking the degradation of SQSTM1/p62 which was required for the delivery of several ubiquitinated cargos to the autophagosome (Additional file 2: Figure S2). Therefore, we chose CQ as the autophagy inhibitor in subsequent experiments. The results showed that SQSTM1/p62 was significantly increased by CQ (Figure 2C-D), and also remarkably increased the secretion of RANTES induced by ox-LDL (Figure 2E-F).

### Berberine activated autophagy in dose- and time-dependence in J774A.1 cells

**Figure 3:**
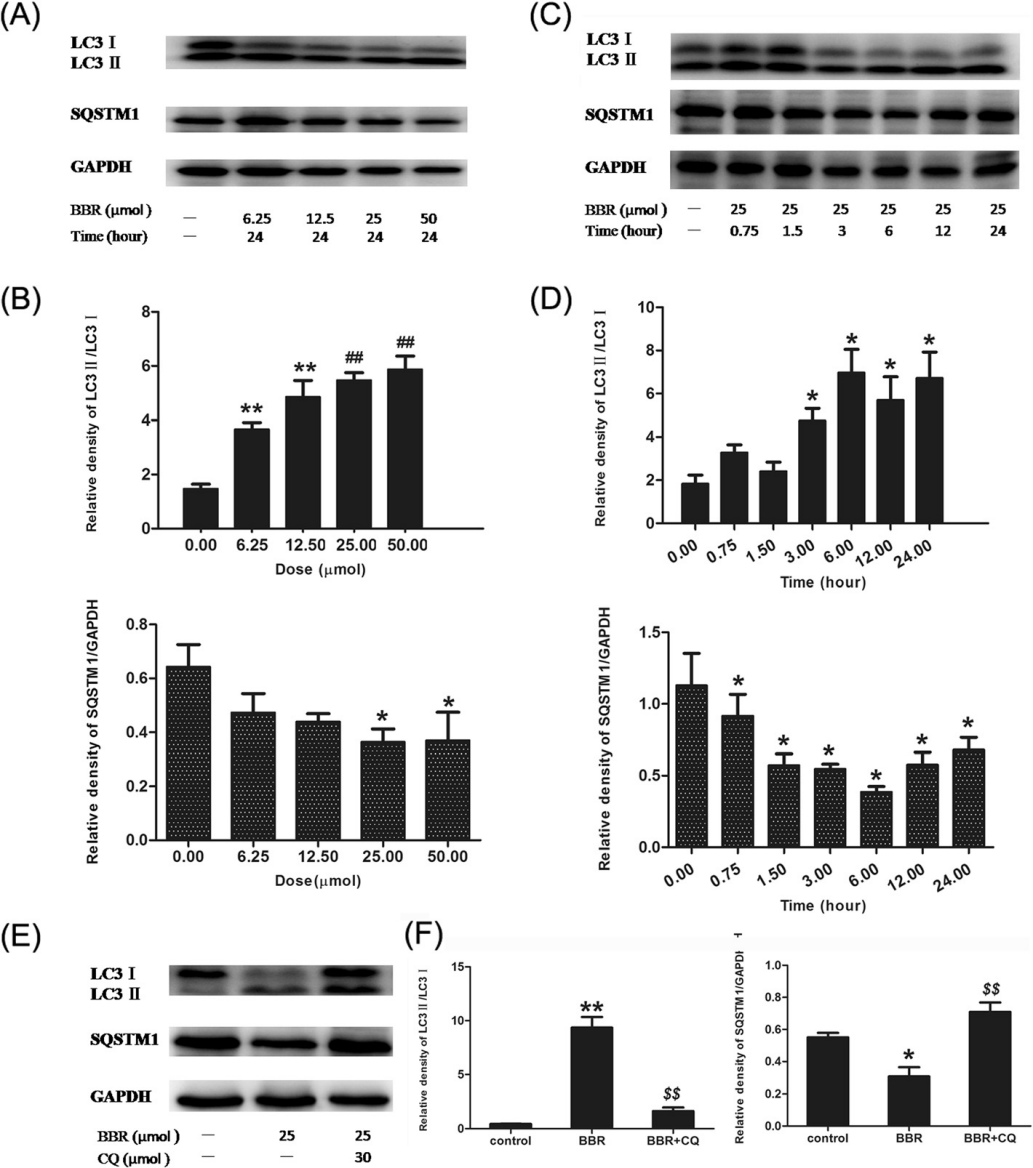
**Berberine induced autophagy in J774A.1 cells. (A)** Representative blots of LC3 and SQSTM1/p62 in J774A.1 cells treated with a series of concentrations of berberine (6.25, 12.5, 25 and 50 μM). **(B)** Bar graphs showed the quantification of endogenous LC3 and SQSTM1. Experiments were repeated at least three times. **(C)** Representative blots of LC3 and SQSTM1/p62 in J774A.1 cells treated with 25 μM berberine for different time intervals (0.75, 1.5, 3, 6, 12 and 24 h). **(D)** Bar graphs showed the quantification of endogenous LC3 and SQSTM1. Experiments were repeated at least three times. **(E)** Representative blots of oLC3 and SQSTM1/p62 in J774A.1 cells treated with 25 μM berberine in the presence or absence of 30 μM CQ. **(F)** Bar graphs showed the quantification of endogenous LC3 and SQSTM1/p62. Data are expressed at the mean ± SEM in the corresponding area. **P < 0.01 V.S. control group, *P < 0.05 V.S. control group; ##P < 0.01 V.S. 6.25 μM and 12.5 μM groups; $$P < 0.01 V.S. BBR group.

To determine whether autophagy could be induced by berberine with a single treatment in J774A.1 cells, we investigated the ratio of LC3II/LC3I and SQSTM1/p62 degradation. Compared with the control, a single treatment with berberine increased the ratio of LC3II/LC3Iand decreased SQSTM1/p62 expression in a dose-dependent (Figure 3A-B) and time-dependent (Figure 3C-D) manner. With the increase in the concentration of berberine, the expression of LC3 was evaluated but SQSTM1/p62 degradation was reduced, especially at concentrations of 25 μM and 50 μM. Furthermore, at different time points (0.75, 1.5, 3, 6, 12 and 24 h) we found that the expression of LC3 was increased at 3, 6, 12 and 24 h whileSQSTM1/p62 degradation was decreased at 0.75, 1.5, 3, 6, 12 and 24 h, with the peak at 6 h. Additionally, the levels of LC3 and SQSTM1/p62 were measured in the presence of CQ to monitor autophagic flux. CQ challenge resulted in further accumulation of SQSTM1/p62 in J774A.1 cells before 24 h-incubation with berberine, compared with that in cells treated with berberine alone (Figure 3E-F). These results suggested that a single-treatment with berberine promoted cellular autophagic process- in J774A.1 cells.

### Berberine prevented ox-LDL-induced inflammation via up-regulation of autophagy in J774A.1 cells

**Figure 4:**
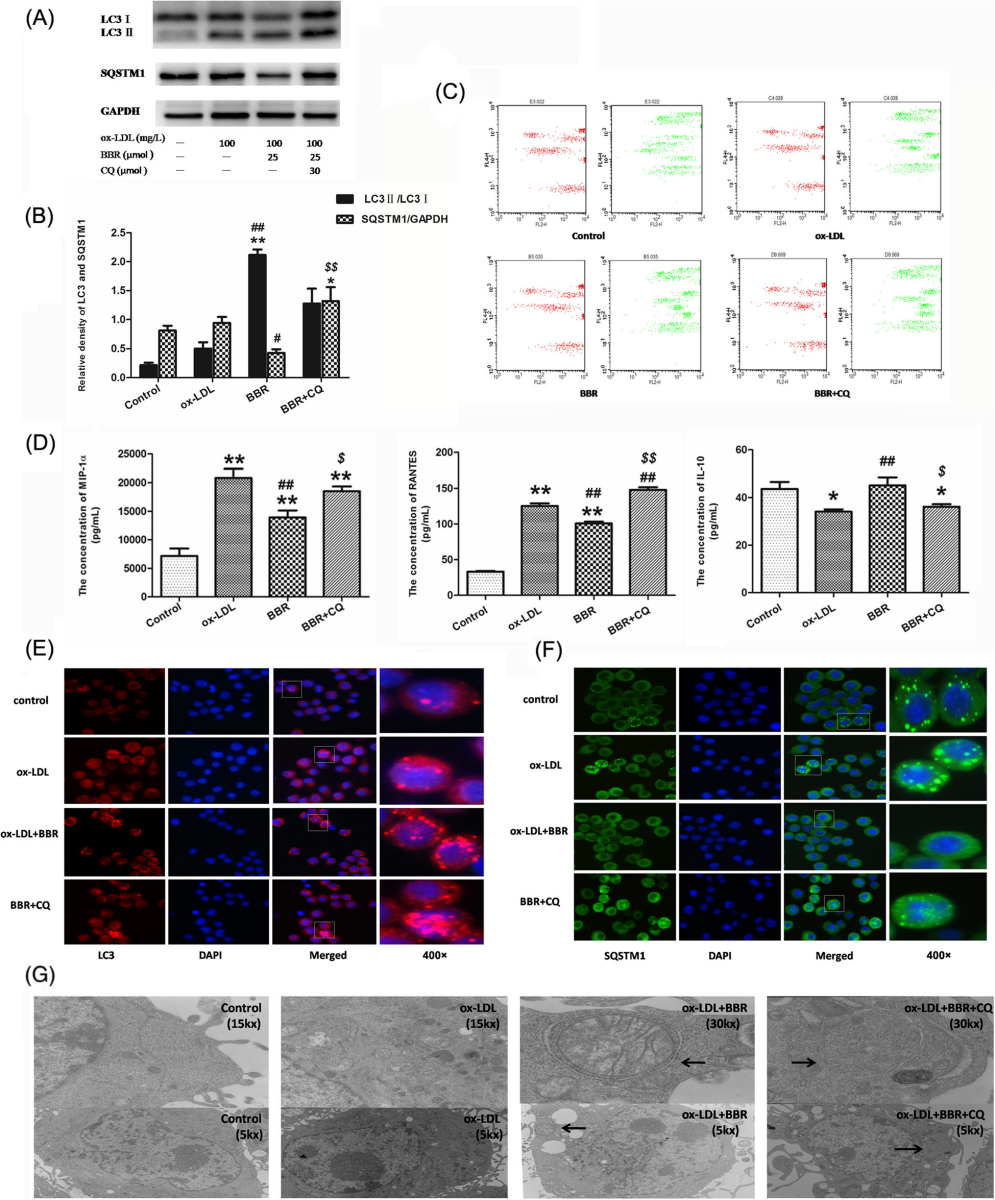
**Berberine attenuated ox-LDL-induced inflammation through the induction of autophagy in J774A.1 cells. (A)** Representative blots of LC3 and SQSTM1/p62 in ox-LDL stimulated J774A.1 cells treated with berberine in the presence or absence of 30 μM CQ. **(B)** Bar graphs showed the quantification of the indicated proteins. **(C)** Representative flow cytometry scatter plot of J774A.1 cells after treatment. Experiments were repeated at least three times. **(D)** The levels of MIP-1α, RANTES and IL-10 in the culture media from ox-LDL-stimulated J774A.1 cells were detected via Aimplex. (n = 4) **(E)** Cells were double stained by LC3 and DAPI and examined by fluorescence microscopy (200×, 400×). **(F)** Cells were double stained by SQSTM1/p62 and DAPI and examined by fluorescence microscopy (200×, 400×). **(G)** Representative TEM images of J774A.1 cells Arrows indicated the autophagosomes (30.0 kx, 15.0 kx, 5.0 kx). **P < 0.01 V.S. control group, *P < 0.05 V.S. control group; ##P < 0.01 V.S. ox-LDL group; $$P < 0.01 V.S. BBR group, $P < 0.05 V.S. BBR group.

To investigate whether berberine could inhibit inflammatory factors through autophagy induction, we tested the change of autophagy and inflammatory factors. Compared with control group or ox-LDL group, berberine significantly increased the ratio of LC3II/LC3I and SQSTM1/p62 degradation (Figure 4A-B). After berberine-induced autophagy was inhibited by CQ, MIP-1α and RANTES notably increased and IL-10 decreased (Figure 4C-D). These results indicated that anti-inflammatory effects of berberine contributed to autophagy in macrophagy cells. To further confirm berberine-induced autophagy in J774A.1 cells stimulated by ox-LDL, immunocytochemistry was utilized to reveal the intracellular localization of LC3 and SQSTM1/p62. Cells in control or ox-LDL group showed little staining of LC3. In contrast, cells treated with berberine or berberine plus CQ displayed a strong positive staining distribution of LC3 (Figure 4E). For SQSTM1/p62, cells treated with berberine plus CQ also showed an intensely positive staining of SQSTM1/p62 (Figure 4F). Ultrastructural changes in cells were examined with transmission electron microscope (TEM). TEM images in control and ox-LDL group displayed normal cytoplasm, characterized by mitochondria, endoplasmic reticulum, free ribosomes and irregular nucleus, as well as few autophagosomes and lysosomes (Figure 4G). In contrast, berberine or berberine plus CQ group dispersedly exhibited many autophagosomes at various stages in cytoplasm. Arrows indicated typical double membrane autophagosomes containing mitochondria and separated cytoplasm.

### Berberine activated autophagy via AMPK/mTOR pathway in J774A.1 cells

**Figure 5:**
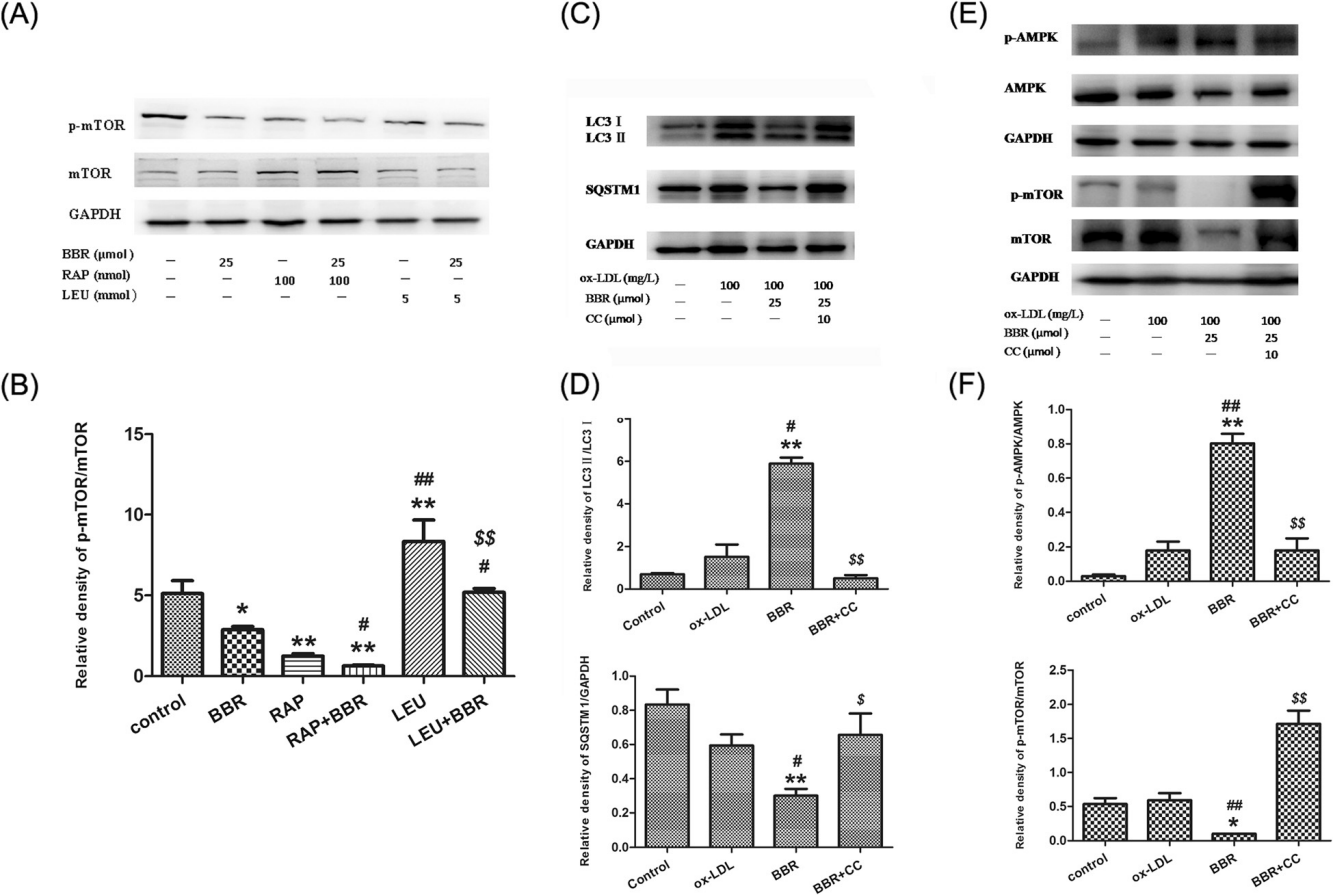
**Berberine induced autophagy in J774A.1 cells was mediated by AMPK/mTOR pathway. (A)** Representative blots of mTOR in BBR treated J774A.1 cells which were incubated with mTOR inhibitor rapamycin (Rap) or mTOR agonist leucine (LEU). **(B)** Bars graphs showed the quantification of indicated proteins. **(C)** Representative blots of LC3 and SQSTM1/p62 in ox-LDL stimulated J774A.1 cells treated with berberine in the presence or absence of 10 μM CC. **(D)** Bars graphs showed the quantification of indicated proteins. **(E)** Representative blots of P-AMPK/AMPK and p-mTOR/mTOR in J774A.1 cells treated with berberine in the presence or absence of 10 μM CC. **(F)** Bar graphs showed the quantification of indicated proteins. Experiments were repeated at least three times. **P < 0.01 V.S. control group, *P < 0.05 V.S. control group; ##P < 0.01 V.S. ox-LDL group, #P < 0.05 V.S. ox-LDL group; $$P < 0.01 V.S.BBR group, $P < 0.05 V.S.BBR group; aa P < 0.01 V.S. BBR group, a P < 0.05 V.S. BBR group; bb P <0.01 V.S.L-L group.

We further explored whether AMPK/mTOR pathway was involved in berberine-mediated autophagy in J774A.1 cells. Firstly, cells were treated with mTOR inhibitor rapamycin (100 nM) or agonist leucine (5 mM) in the presence or absence of 25 μM BBR. As the Figure 5A-B shown, mTOR inhibitor rapamycin suppressed the amount of LC3 II/I which was down-regulated more with the treatment of BBR in the presence of rapamycin. Conversely, leucine enhanced the level of LC3 II/I which was also down-regulated by exposure of BBR. Obviously, the mTOR was involved in the cellular response to berberine. In addition, cells were treated with ox-LDL for 1 h in the presence or absence of 10 μM compound C, an AMPK inhibitor, followed by the treatment with berberine for another 24 h. We detected that berberine-induced expression of LC3 and SQSTM1/p62 degradation were blocked by pretreatment with compound C (Figure 5C-D). At last, berberine induced an obvious AMPK activation which was neutralized by compound C. Meanwhile, berberine decreased the ratio of p-mTOR (Ser2448)/mTOR (Figure 5E-F). It is indicated that AMPK/ mTOR signaling pathway may contribute to berberine-induced autophagy.

## Discussion

AS is a seriously worldwide health concern and oxLDL-induced vascular endothelial injury is a driving force in its initiation and development [15-17]. Berberine, a natural compound from *Coptis chinensis Franch*, has been used for preventing cardiovascular diseases [18,19], especially oxLDL-induced macrophage injuries [20-22]. However, the molecular mechanisms are not fully understood. In our study, we demonstrated that berberine attenuated oxLDL-induced inflammatory factors by stimulation of macrophage autophagy via the AMPK/mTOR signaling pathway.

Autophagy is an essentially metabolic process in which damaged or senescent organelles can be removed and thus basal energy balance can be maintained [23]. Dysregulation of autophagy might result in many diseases including AS [24]. The protective actions of autophagy in AS have been shown by some studies [25,26]. Under normal circumstances, increased autophagosome formation, which is characterized by increases in LC3-II conversion, occurs during cellular autophagy induction in cells. LC3, an autophagy protein marker, is converted from cytosolic LC3-I into enzymatic LC3-II when autophagy is activated; thus, the ratio of LC3-II to LC3-I can be considered as a standard marker for the detection of autophagy [27,28]. The conversion of soluble LC3-I to lipid bound LC3-II is associated with the formation of autophagosomes and also the amount of LC3-II reflects the number of autophagosomes [29]. Some researchers showed that the conversion of LC3-I to LC3-II does not necessarily result in complete autophagy [30]. In the later stage of autophagy, LC3-II may degrade by SQSTM1/p62 which means a complete autophagy [29]. Moreover, autophagosome formation increases autophagic flux, which caused elevation of p62/SQSTM1 protein degradation [31]. To verify whether berberine could regulate the complete autophagy, we tested the changes of LC3-II/LC3-I ratio and of p62/SQSTM1 amount. In our studies, we first detected that berberine decreased inflammation via autophagy-dependent mechanism, which was demonstrated by the increased ratio of LC3II/LC3I and SQSTM1/p62 degradation as well.

In accordance with previous studies [32,33], 100 mg/L ox-LDL can result in autophagy in J774A.1 cells. It had been showed that ox-LDL treatment intensified autophagy in human umbilical vein endothelial cells. The up-regulation of both LC3-II and beclin-1 proteins reached two peaks at 0.5 h and 6 h, and declined at 24 h and 48 h, respectively. While we found that the expression of LC3II/I induced by ox-LDL did not increase until 12 h and then decreased in J774A.1 cells. The different changes of time points may be due to different cell types. Our results indicated that ox-LDL-induced autophagy was time-dependent which could become dysfunctional in advanced stages of AS. Autophagy deficiency promotes AS partially in part through oxidative damage [34] and inflammation factors release [7]. Furthermore, our results showed that the autophagy induced by ox-LDL was decreased further at 12 h and 24 h, which was consistent with the former report.

It was reported that suppression of autophagy activated nuclear factor kappa-B (NF-κB) signaling and then induced TNF-α and IL-6 in THP-1 cells [35]. Our studies also confirmed that inflammatory factors were increased after autophagy was inhibited by chloroquine (CQ). Berberine induced autophagy in the way of dose- and time-dependent manners in J774A.1 cells. At 24 h, berberine decreased expression of MIP-1α and RANTES, and increased expression of IL-10, which was prevented by CQ treatment. After p62/SQSTM1 degradation was inhibited, the inflammatory factors, MIP-1α and RANTES, increased but IL-10 decreased. Consequently, berberine may inhibit inflammation via autopahgy pathway. Another study showed that the beneficial autophagic process reduced TNF-induced inflammation and protected the endothelial cells [36]. Our data also proved this beneficial autophagic process induced by berberine which might partially inhibited ox-LDL-induced inflammation reaction in macrophages.

Furthermore, we investigated the potential molecular mechanisms by which berberine induced autophagy. AMPK/mTOR is the essential regulator of cellular autophagy. AMPK is required for the protective effects of berberine in cardiovascular diseases [37]. Berberine significantly increased AMPK activity via reactive oxygen species (ROS) production and knockdown of AMPKα1 abolished the effect of berberine [38]. The mammalian target of rapamycin (mTOR) kinase is a central inhibitor of autophagy. mTOR consist of mTOR complex 1 (mTORC1) and mTOR complex 2 (mTORC2). mTORC1 is rapamycin sensitive and acts as a major checkpoint that coordinate the balance between cell growth and autophagy [39]. Rapamycin, a mTOR inhibitor, suppressed the amount of LC3 II/I which was downregulated by the treatment of BBR in the presence of rapamycin. Conversely, leucine enhanced the level of LC3 II/I which was also down-regulated by exposure of BBR. Obviously, the mTOR was involved in the cellular response to berberine. We found that pretreatment with compound C increased the expression of p-mTOR/mTOR, and inhibited LC3 expression and p62/SQSTM1 degradation in oxLDL-stimulated J774A.1 cells. This result suggested that AMPK might be an upstream factor of mTOR, and berberine-induced autophagy may be preceded by the activation of AMPK/mTOR in J774A.1 cells. Therefore, our data demonstrated that the AMPK/mTOR signaling pathway may be involved in berberine-induced autophagy in macrophage, although the activation of AMPK/mTOR pathway induced by berberine has been reported in other cell lines [40,41], our data demonstrated that the AMPK/mTOR signaling pathway may be involved in autophagy induced by berberine in macrophage. In addition, as displayed in Additional file 3: Figure S3, the mRNA expressions of mTOR was consistent with the protein levels which indicated that the BBR regulated AMPK/mTOR through transcriptional mechanism.

Additionally, the AMPK/SIRT1 signaling pathway exerted protective role for AS via autophagy induction [34,36]. Berberine increased the expression of SIRT1, a regulator of autophagy, at both the protein and mRNA levels in macrophages in a dose-dependent manner [42]. However, whether berberine could induce autophagy through the AMPK/SIRT1 signaling pathway in macrophagy cells needs further investigation.

## Conclusions

Our results elucidate potential molecular mechanisms of the anti-inflammatory effects of berberine, in which autophagy may play an important protective role. Furthermore, the AMPK/mTOR signaling pathway may contribute to the regulation of berberine-induced autophagy.

## Additional files

Additional file 1: Figure S1. Representative flow cytometry scatter plot of the different concentrations of ox-LDL(0, 25, 50, 100,150, 200, 300 mg/L) for 24h in J774A.1 cells. Additional file 2: Figure S2. The effect of 3-MA and CQ on J774A.1 cell lines. Additional file 3: Figure S3. Quantitative PCR analysis of mTOR mRNA levels in ox-LDL induced J774A.1 cells treated with BBR, BBR and CC for 24h.

## Abbreviations

AS: Atherosclerosis
Ox-LDL: Oxidized low density lipoprotein
BBR: Berberine
MIP-1α: Inflammatory protein 1α
RANTES: Regulated upon activation normal T cell expressed and secreted
IL-10: Interleukin-1
AMPK: Adenosine 5’-monophosphate-activated protein kinase
mTOR: Mammalian target of rapamycin
3-MA: 3-methyladenine
CQ: Chloroquine
CC: Compound C
DAPI: 4′, 6-diamidino-2-phenylindole
Rap: Rapamycin
LEU: Leucine
