# Supplementary material for: Berberine alleviates ox-LDL induced inflammatory factors by up-regulation of autophagy via AMPK/mTOR signaling pathway
Source: J Transl Med. 2015 Mar 15;13:92. doi: 10.1186/s12967-015-0450-z (PMC4365560; doi:10.1186/s12967-015-0450-z)
Supplement: Additional file 1: Figure S1. — Representative flow cytometry scatter plot of the different concentrations of ox-LDL(0, 25, 50, 100,150, 200, 300 mg/L) for 24h in J774A.1 cells. [file 12967_2015_450_MOESM1_ESM.pdf]

Ox-LDL: 0mg/l

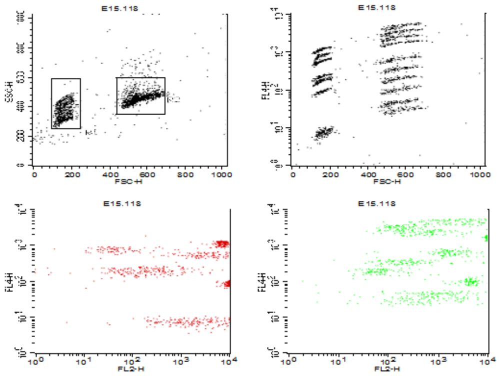

Ox-LDL:25mg/l

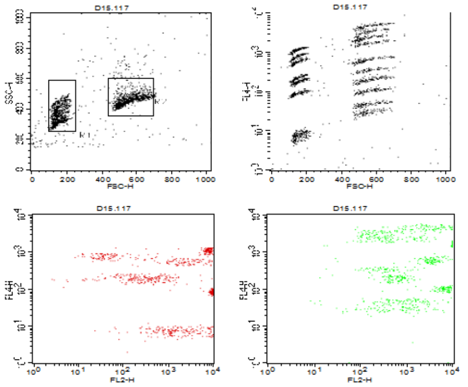

Ox-LDL: 50mg/l

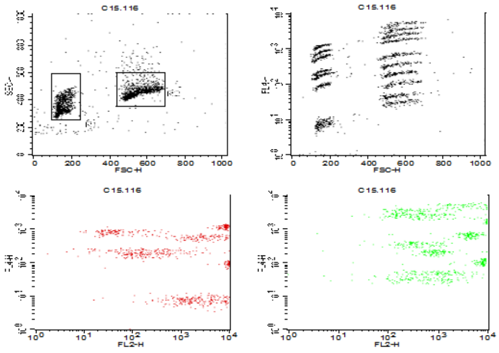

Ox-LDL: 100mg/l

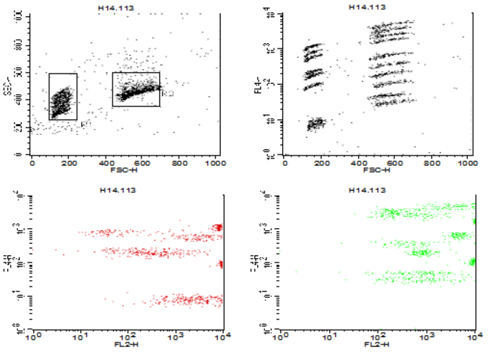

Ox-LDL: 150mg/l

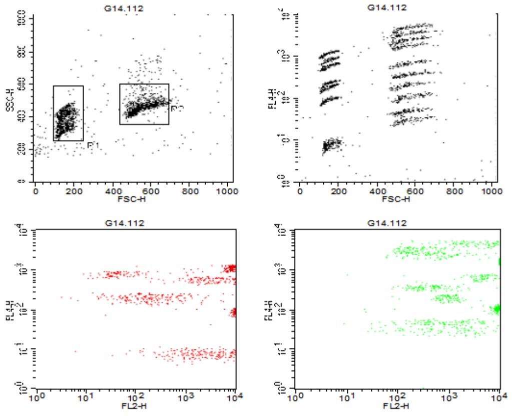

Ox-LDL: 200mg/l

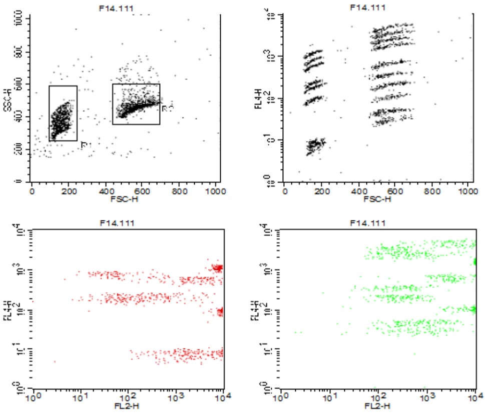

Ox-LDL: 300mg/l

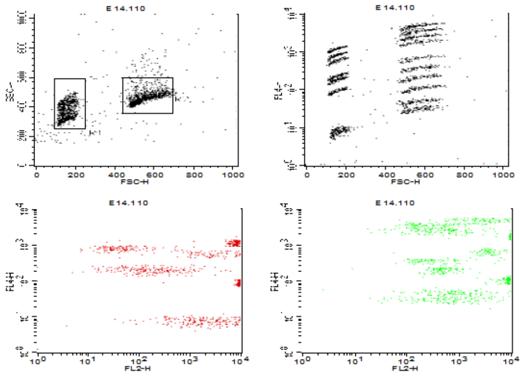

Representative flow cytometry scatter plot of the different concentrations of ox-LDL(0, 25, 50, 100,150, 200, 300 mg/L) for 24h in J774A.1 cells. The levels of MIP-1a, RANTES, IL-10 in the medium were detected via AimPlex technology. For each concentration, the upper left panel: A dot plot with FSC-H(forward scatter-high, X-axis) and SSC-H (side scatter-high, Y-axis) in linear display mode. The fluorescence intensity of PE (Phycoerythrin) on the horizontal axis and the APC (Allophycocyanin) fluorescence intensity on the vertical. Create Gate 1(R1) for the smaller (4 micron size, S4) beads and Gate 2(R2) for the larger (5 micron size, S5) beads. The upper right panel: All bead populations are clearly separated on the histograms and dot plots through adjusting APC. Each bead population conjugated with a specific capture antibody to trap the protein of interest.The different bead population represents different cytokines. The amount of the analyte captured is detected via a biotinylated antibody against a secondary epitope of the protein, followed by a streptavidin-R-phycoerythrin (streptavidin-PE) treatment.The fluorescent intensity of PE on the beads is quantified on a flow cytometer. Apply proper "APC" - %PE color compensation: Red represents gate1 (4 micron size beads) and green gate 2 (5 micron size beads) (lower left panel-lower right panel). Size 4 micron, Peak #6(S4P6) is IL-10, Size 4 micron, Peak #9(S4P9) is anti-mouse MIP-1α and Size 5 micron, Peak #7 (S5P7) is RANTES.
