# Supplementary material for: Berberine alleviates ox-LDL induced inflammatory factors by up-regulation of autophagy via AMPK/mTOR signaling pathway
Source: J Transl Med. 2015 Mar 15;13:92. doi: 10.1186/s12967-015-0450-z (PMC4365560; doi:10.1186/s12967-015-0450-z)
Supplement: Additional file 2: Figure S2. — The effect of 3-MA and CQ on J774A.1 cell lines. [file 12967_2015_450_MOESM2_ESM.pdf]

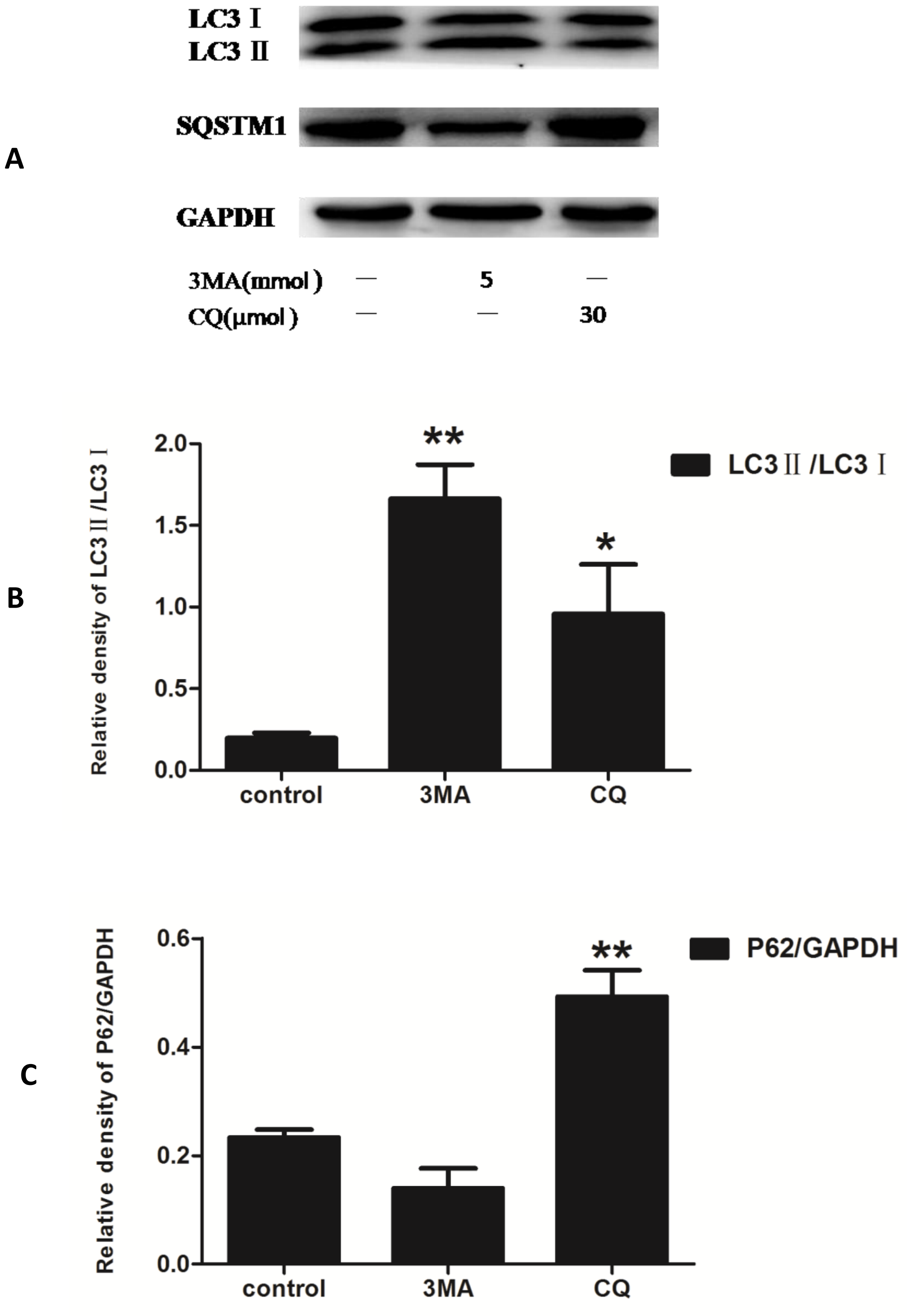

The effect of 3-MA and CQ on J774A.1 cell lines. (A) Representative blots of LC3 and SQSTM1/p62 in J774A.1 cells treated with 3-MA (5mmol/L)and CQ (30 μm),separately for 24h. Bar graphs showed the quantification of endogenous LC3 **(B)** and SQSTM1/p62 **(C)**. Experiments were repeated at least three times. **P<0.01 V.S. control group, *P<0.05 V.S. control group; * *P<0.01.
