# Supplementary material for: Berberine alleviates ox-LDL induced inflammatory factors by up-regulation of autophagy via AMPK/mTOR signaling pathway
Source: J Transl Med. 2015 Mar 15;13:92. doi: 10.1186/s12967-015-0450-z (PMC4365560; doi:10.1186/s12967-015-0450-z)
Supplement: Additional file 3: Figure S3. — Quantitative PCR analysis of mTOR mRNA levels in ox-LDL induced J774A.1 cells treated with BBR, BBR and CC for 24h. [file 12967_2015_450_MOESM3_ESM.pdf]

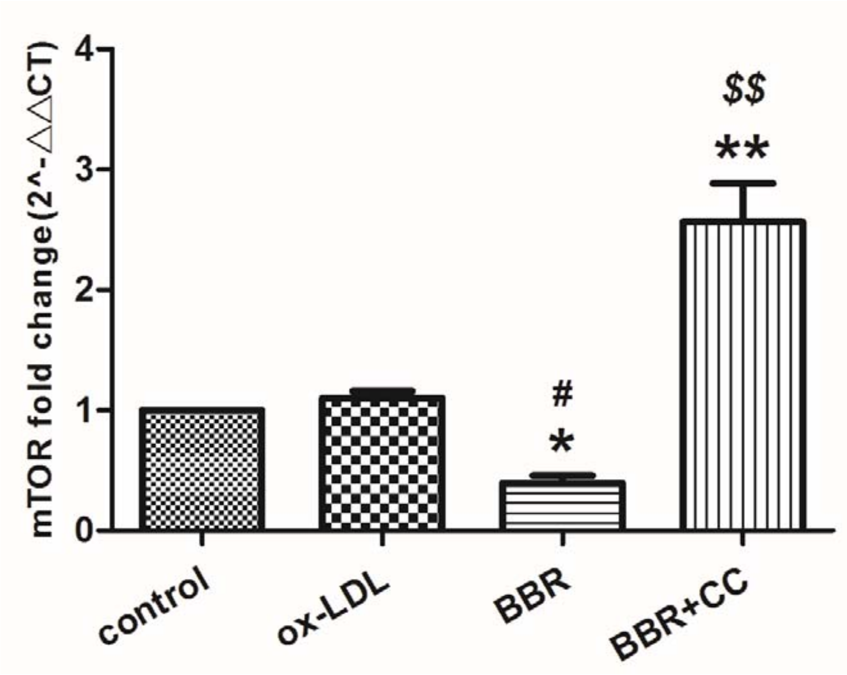

Effect of berberine on mRNA level of mTOR. Quantitative PCR analysis of mTOR mRNA levels in ox-LDL induced J774A.1 cells treated with BBR, BBR and CC for 24h. Total mRNA was isolated and mTOR mRNA was quantified by real-time PCR compared with GAPDH. The PCR primers for mTOR were 5′-GCAGATTTGCCAACTACC -3′ and 5′ - CACGGAGAACGAGGACA -3′. The PCR primers for GAPDH which used as endogenous control were 5′-GGTGAAGGTCGGTGTGAACG -3′ and 5′ - CTCGCTCCTGGAAGATGGTG -3′. Bar graphs showed the quantification of mRNA level of mTOR. Experiments were repeated at least three times. **P<0.01 V.S. control group, *P<0.05 V.S. control group; ##P<0.01 V.S. ox-LDL group, #P<0.05 V.S. ox-LDL group; $$P <0.01 V.S.BBR group.
